# Mannose-Binding Lectin Reduces Oxidized Low-Density Lipoprotein Induced Vascular Endothelial Cells Injury by Inhibiting LOX1-ox-LDL Binding and Modulating Autophagy

**DOI:** 10.3390/biomedicines11061743

**Published:** 2023-06-17

**Authors:** Xuelian Zhou, Xuefeng Chen, Li Zhang, Jinna Yuan, Hu Lin, Mingqiang Zhu, Xiaoqin Xu, Guanping Dong, Junfen Fu, Wei Wu

**Affiliations:** Department of Endocrinology, Children’s Hospital, Zhejiang University School of Medicine, National Clinical Research Center for Child Health, 3333 Binsheng Road, Hangzhou 310052, China; 6512031@zju.edu.cn (X.Z.);

**Keywords:** mannose-binding lectin, endothelial cells injury, autophagy, LOX1, atherosclerosis

## Abstract

**Objective:** To investigate the role of mannose-binding lectin (MBL) in modulating autophagy and protecting endothelial cells (ECs) from oxidized low-density lipoprotein (ox-LDL)-induced injury. **Methods:** Serum MBL concentration and carotid intima-media thickness (cIMT) were measured in 94 obese and 105 healthy children. ECs were transfected with MBL over-expression plasmid, LOX1 was knocked-down to explore the protective role of MBL in ox-LDL induced ECs injury. Dendritic cells (DCs) were co-cultured with ECs, and inflammatory factors, DC maturation, and autophagy was assessed. WT and ApoE^−/−^ mice were fed with a high fat diet (HFD) with or without MBL-adenovirus injection for 16 weeks and aortic vascular endothelial tissue was isolated, then atherosclerotic plaque, cell injury and autophagy were analyzed. **Results:** Serum MBL concentration in obese children was lower than healthy controls and was negatively correlated with cIMT. The uptake of ox-LDL was decreased in LOX1 knock-down ECs. MBL over-expression in vitro inhibited LOX1-ox-LDL binding. Both LOX1 knock-down and MBL over-expression can ameliorate EC autophagy and cell injury. MBL over-expression in vivo alleviated atherosclerotic plaque formation, influenced DC maturation and down-regulated IL-6, IL-12, and TNF-a levels. **Conclusions:** MBL exerts a protective role in ox-LDL-induced EC injury by modulating DC maturation and EC autophagy via inhibiting LOX1-ox-LDL binding.

## 1. Introduction

Atherosclerosis and subsequent cardiovascular diseases (CVDs) contribute to the leading causes of mortality worldwide [1,2]. Although they benefit from optimal medical and pharmaceutical care, a significant portion of CVD patients still suffer from recurrent cardiovascular events [3]. Increasing evidence suggested that the immune system’s activation and the inflammatory response have a crucial role in the initiation and progression of atherosclerosis, rather than just lipid deposition [4,5,6]. Canakinumab, a human monoclonal IL-1β antibody, was confirmed to have a beneficial effect for atherosclerosis in the phase 3 CANTOS trail [7]. However, immune-modulators such as tocilizumab (humanized anti-IL-6R antibody) have been shown to have a protective effect in non-ST-elevation myocardial infarction patients while still posing the risk of increasing circulating levels of cholesterol and triglycerides [8,9]. Therefore, selecting an appropriate immunoregulation target along with an optimal treatment window is urgently needed.

Ox-LDLs, as an important atherosclerosis contributor, were mainly up taken by ECs through binding with lectin-like oxidized low-density lipoprotein receptor-1 (LOX-1), mediating immune reactions and participating in the pathogenesis of atherosclerosis [10,11]. A study revealed that ox-LDL concentration polarization might activate autophagy and apoptosis via the LOX-1 pathway [12], while over-expression of LOX-1 may attenuate ECs protective autophagy and induce apoptotic death of bovine aortic ECs [13]. Thus, autophagy level might relate to the outcome of CVDs. MBL, a member of C-type lectins family, acts as opsonin in innate immunity, which can activate the complement system by selectively binding to mannan-rich pathogens and can also trigger opsonophagocytosis through direct binding to cell surface receptors. Several studies have shown that high levels of MBL are harmful to patients with ischemic stroke, diabetic nephropathy, and retinopathy [14,15,16]. However, studies on MBL genotypes revealed that low or deficient MBL was a risk factor for developing CVDs [17,18]. Other studies reported both low and high levels of MBL, indicating the susceptibility for CVDs in T2DM patients [19,20]. Early onset obesity is associated with an increased risk of CVDs in adulthood; however, the correlation between MBL and cardiovascular risk in children with obesity has not been reported. This study found that blood MBL level decreased in children with moderate to severe obesity and was negatively correlated with cIMT. Thus, whether MBL has a protective or harmful effect in CVDs, as well as relevant underlying mechanisms, remains unclear.

MBL shares similar domains with LOX1, and a previous study confirmed that MBL could bind to ox-LDL in vitro [21]. In this study, we aim to investigate whether MBL competitively inhibits the binding of ox-LDL and LOX1, and thus protects ECs from ox-LDL-induced cell injury. The roles of DCs and autophagy were also illustrated.

## 2. Materials and Methods

### 2.1. Diagnosis Criteria and Sample Collection

A total of 94 children with obesity (Obese group) and 105 age-matched healthy controls (Control group) were enrolled in the study between July 2019 and Aug 2020 in the Children’s Hospital of Zhejiang University, School of Medicine. Children with obesity came to the endocrinology department for metabolic assessment, while the control group came for regular health examinations. Children with BMI exceeding the 95th percentile of their age were diagnosed as obesity; for the healthy controls, the BMI was less than the 85th percentile, and BMI was calculated as dividing weight (kg) by height squared (m^2^). Exclusion criteria: (1) obesity secondary to medications, hypothalamic surgery, and genetic obesity syndromes, (2) participants with systemic or organic diseases, (3) participants with malnutrition, (4) recent medication which may affect metabolism, such as antidiabetic drugs, antihypertensive drugs or lipid-lowering drugs, etc.

All of them underwent detailed history and physical examination by trained pediatric endocrinologists. B-ultrasound (Philips 5500) was used to evaluate the cIMT. Fasting blood samples were collected, and supernatants were centrifuged at 1000× *g* for 10 min at 4 °C for MBL concentration determination (Human MBL Quantikine ELISA Kit, R&D, Massachusetts, USA).

### 2.2. Cells Culture

Mouse ECs and DCs were purchased from Tongpai (Shanghai, China) and cultured in RPIM-1640 medium (containing 10% fetal bovine serum) at 37 °C and 5% CO_2_. Mouse spleen-derived DCs were prepared according to the previously described method [22]. Flow cytometer was used to identify DCs with biomarkers of CD 11c (Bioss, Beijing, China) and CD80 (Biolegend, San Diego, CA, USA).

ECs were treated with 0, 100, or 200 μg/mL ox-LDL (Yiyuanbiotech, Guangzhou, China) for different time durations (0, 24, 48 h). Autophagy inhibitor Chloroquine (25 μM, TCI, Shanghai, China) and autophagy inducer rapamycin (0.02 μg, Gene operation, Michigan, USA) were pretreated for 1 h and then treated with 100 μg/mL ox-LDL for 24 h, then autophagosome marker LC3-II, Lamp2a and cell damage marker ET-1 were detected to explore the role of autophagy in ox-LDL-induced ECs injury.

LOX1-siRNA and MBL over-expression plasmid were constructed (for details see Section 2.3) to explore the role of MBL in ox-LDL-induced ECs injury. Mannan can competitively combine with MBL; thus, ECs were pretreated with mannan (6 mg/L, Aladdin, Shanghai, China) for 48 h and then treated with 100 μg/mL ox-LDL for 24 h to explore the role of MBL in the regulation of autophagy in ox-LDL-treated ECs.

DCs were co-cultured with ECs, and then treated with ox-LDL; the maturation of DCs, and inflammatory factors IL-6, IL-12, and TNF-a were measured to investigate whether DCs are involved in the protective effect of MBL against ox-LDL-induced EC injury.

### 2.3. Cell Transfection

Small interfering RNA (siRNA) against LOX1 (LOX1-siRNA, Forward: 5′-ACTCTGGTCATCCTCTGCCT-3′, Reverse: 5′-CCTGCTGAGTAAGGTTCGCT-3′) or negative control siRNA (Forward: 5′-CTCTCTGCTCCTCCCTGTTC-3′, Reverse: 5′-AAATCCGTTCACACCGACCT-3′), LC3-GFP plasmid, MBL overexpression plasmid (MBL plasmid), control plasmid, MBL overexpression adenovirus, and an empty vector were synthesized by Genepharma (Shanghai, China). Lipofectamine 2000 reagent (Invitrogen, Waltham, MA, USA) was used for transfection of oligonucleotides or vectors according to the standard protocol.

### 2.4. Cells Viability Assay

A 300 μL ECs suspension (4 × 10^4^ cells/well) was incubated with different concentrations of ox-LDL for 24 h and 48 h. Then, CCK-8 reagents (30 μL/well) (Beyotime, Shanghai, China) were added into each well for 2 h. The absorbance was measured at 450 nm with a microplate reader.

### 2.5. Cell Apoptosis Assay

Flow cytometry was used to detect EC and DC apoptosis rates. After treatment with ox-LDL for 24 h, ECs and DCs were resuspended with binding buffer, followed by staining with 5 μL annexin V-FITC (KeyGen Biotech, Nanjing, China) and 10 μL PI (Sigma, St. Louis, MO, USA). The apoptotic cells were analyzed by FlowJo software (Tree Star, Ashland, OR, USA) version 10.0.8.

### 2.6. DCs Maturation Assay

DCs maturation rates were analyzed by flow cytometry with anti-CD 80 and CD86 antibody (eBioscience, San Diego, CA, USA). A volume of 200 µL of DCs (5 × 10^4^/mL) were incubated at 37 °C and 5% CO_2_ for 24 h, then the upper compartment cells were collected and washed twice with PBS (containing 2% FBS); the supernatant was discarded, and 95 μL PBS and 5 μL corresponding antibodies were added. Next, they were swirled and mixed, and incubated at 4 °C for 30 min in darkness, after which 1 mL PBS was added for centrifuge washing for 2 times. The maturation rate was analyzed by FlowJo software (Tree Star, Ashland, OR, USA) version 10.0.8.

### 2.7. Enzyme-Linked Immunosorbent Assay (ELISA)

Molecular marker of ECs damage from the supernatants were detected by mouse ET-1 ELISA kit (Cloud-Clone, Wuhan, China). Inflammatory factors IL-6, IL-12, and TNF-a levels from the supernatants were detected by IL-6 ELISA kit (ExCell Bio, Shanghai, China), IL-12 p70 ELISA kit (ExCell Bio, Shanghai, China), and TNF-a ELISA kit (ExCell Bio, Shanghai, China). Operations were performed according to the manufacturer’s instructions, and the absorbance was detected at 450 nm wavelength.

### 2.8. Quantitative Real-Time Polymerase Chain Reaction (qRT-PCR)

Cell total RNAs were prepared with Trizol reagent (Invitrogen, Waltham, MA, USA). qPCR was performed using an SYBr green Pcr kit (Thermo, Waltham, MA, USA). The gene expression levels were quantified with a 7900 Fast Real-Time PCR instrument according to the instruction. Primers used in this experiment were as follows: LOX1 forward: 5′-ACTCTGGTCATCCTCTGCCT-3′, reverse: 5′-CCTGCTGAGTAAGGTTCGCT-3′. MBL forward: 5′-TGCTTACCCAGGCAAGCCTGTG-3′, reverse: 5′-TGCTTACCCAGGCAAGCCTGTC-3′.

### 2.9. Western Blot

Proteins were homogenized in radioimmunoprecipitation assay buffer (Beyotime, Shanghai, China), then quantified by the bicinchoninic acid (BCA) protein assay kit (Beyotime, Shanghai, China). Immunoblot assays were performed using primary antibodies against LC3 (1:1000, Abgent, San Diego, CA, USA), MBL antibody (1:100, Abcam, Cambridge, UK), LOX1 antibody (1:1000, Abcam, Cambridge, UK), and β-actin (Abcam, Cambridge, UK) overnight at 4 °C. Following this, the blots were washed in TBS-T and incubated with HRP-conjugated secondary antibody (Jackson111-095-003, Pennsylvania, USA) for 1 h at room temperature. Image J (National Institutes of Health, Bethesda, MD, USA; version 1.48) was used for semi-quantitative analysis.

### 2.10. Immunofluorescence

LC3-GFP plasmid was transfected into ECs, and 48 h later was treated with ox-LDL for 24 h, then fixed in 4% formaldehyde for 10–15 min and blocked with 5% bovine serum albumin (BSA) within 1% Triton X-100 for 5–10 min at room temperature (SenBeiJia Biological Technology, Nanjing, China). Next, it was incubated with Lamp2a primary antibody (1:200, Abcam, Cambridge, UK) at 4 °C overnight, after which the mixture of fluorescent secondary antibody and DAPI were diluted at a concentration of 1:500 (Boster Biological Technology, Wuhan, China), and incubated at room temperature in the dark for 30–60 min. Finally, a laser confocal microscope was used to take photos (ZEISS, Oberkochen, German).

After the aortic vascular endothelial tissue was dehydrated, rendered transparent, and paraffin-embedded, serum was added to block antibodies at room temperature for 30 min. The blocking fluid was shaken off and incubated with primary antibody CD31 (1:100, Abcam, Cambridge, UK), CD11C (1:100, Abcam, Cambridge, UK), LC3-II (1:100, Proteintech Group, Wuhan, China), and LOX1 (1:100, Abcam, Cambridge, UK) at 4 °C overnight. Slices were then rinsed with PBS 3 times, after which diluted fluorescent secondary antibody were added and incubated in a wet box at 20–37 °C for 1 h and rinsed with PBS 4 times. DAPI (1:500, Boster Biological Technology, Wuhan, China) were added and incubated at room temperature in the dark for 5 min. The slices were sealed with an anti-fluorescence quenching agent (SouthernBiotech, 0100-01, Birmingham, USA), after which the images were collected under a fluorescence microscope (Olympus, BX53).

### 2.11. Co-Immunoprecipitation (Co-IP) Analysis

Co-IP analysis was used to confirm MBL can inhibiting LOX1-ox-LDL binding. The treated cells were collected, and proteins were extracted. An ultraviolet spectrophotometer was used to determine the protein concentration. Then, 20 μL of Protein A/G agarose beads, 180 μL of PBS, and MBL antibody at a ratio of 1:100 (Abcam, Cambridge, UK) was mixed and incubated at room temperature for 1.5 h with rotation. Samples were centrifuged at 4 °C for 2 min at 2000 rpm/min and washed with PBS 3 times, after which the 400 μL (1 mg) protein lysate was added, and the samples were rotated and incubated at 4 °C for 2 h. After centrifugation at 2000 rpm/min at 4 °C for 2 min, they were washed with PBS 3 times, then the precipitation was collected and added into 1 × Loading Buffer and boiled for 5 min. The supernatant was collected after centrifugation at 12,000 r/min at 4 °C for 5 min and then prepared for the Western Blot experiment (see Section 2.9).

### 2.12. Animals and Ex Vivo Experiment

ApoE^−/−^ mice and C57BL/6J mice (6 weeks, male) were purchased from Vital River Laboratory Animal Technology Co. (Beijing, China). All the animals were housed in an environment with a temperature of 22 ± 1 °C, a relative humidity of 50 ± 1%, and a light/dark cycle of 12/12 h. All animal studies (including the mice euthanasia procedure) were done in compliance with the regulations and guidelines of Zhejiang University institutional animal care and conducted according to the AAALAC and the IACUC guidelines.

To investigate whether MBL can reduce ox-LDL-induced EC injury by inhibiting LOX1-oxLDL binding and modulating autophagy in vivo, HFD-induced atherosclerosis mice models and MBL over-expression plasmid were constructed. Mice were divided into four groups, WT mice + 16 weeks’ HFD, ApoE^−/−^ mice + 16 weeks’ HFD, ApoE^−/−^ mice + 16 weeks’ HFD + intraperitoneal chloroquine injections every day, and ApoE^−/−^mice + 16 weeks’ HFD + MBL-adenovirus injected retro-orbital every four weeks. Sections of thoracic aortic vascular endothelial tissue were collected, and stained with Oil Red O, HE, and TUNEL staining (TUNEL apoptosis assay kit, Roche Applied Science, Basel, Switzerland), and the immunohistochemical results were used to evaluate the formation of atherosclerotic plaques, the expression of LOX1, uptake of ox-LDL, the apoptosis rate of ECs, and the expression of LC3-II. Immunofluorescence was viewed under a fluorescence microscope (Olympus BX53).

### 2.13. Statistical Analysis

Statistical analysis of the data was performed using GraphPad Prism software 8.0. Normal distributed data were presented as means ± SD, and a multiple t test was used to analyze individual differences. Non-normally distributed data were presented as median (25th percentile, 75th percentile), and the Mann–Whitney U test was used to analysis the differences between groups. Spearman correlation analysis was performed to evaluate the correlation between serum MBL concentration and cIMT. Data were collected from at least 3 independently experiments both in vivo and vitro, and *p* < 0.05 was statistically significant.

## 3. Results

### 3.1. Serum MBL Level Decreased in Children with Obesity and Negatively Correlated with cIMT

**Figure 1:**
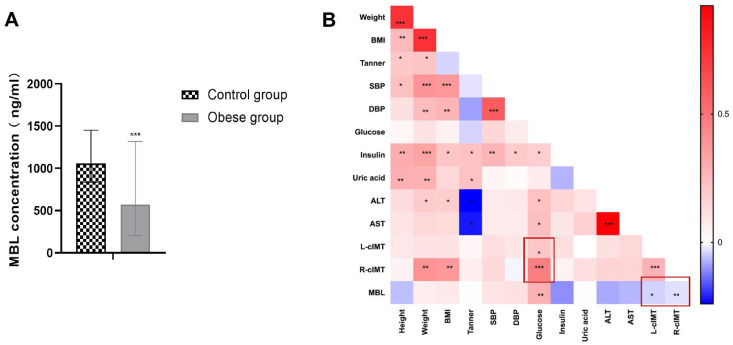
**Serum MBL concentration and the correlation with cIMT.** (**A**) Serum concentration of MBL in children with obesity and healthy controls: serum MBL in 94 obese and 105 healthy children were measured by ELISA kit (R&D), the data showed MBL concentration in the obese group was significantly lower than that of control group. (**B**) Heat map of correlation between physical parameters and laboratory findings: physical parameters and laboratory parameters of the participants were collected to explore the risk factor cIMT. Among all the parameters, blood glucose was positively correlated while serum MBL concentration was negatively correlated with cIMT; see the red frame in Figure 1B. * *p* < 0.05, ** *p* < 0.01, *** *p* < 0.001.

In total, 94 obese and 105 healthy children were enrolled into this study. The details of the participants are listed in Supplementary Table S1. There was no difference in the average age in the two groups. The serum concentration of MBL was 573 (204, 1381) ng/mL in the obese group, and 1057 (838, 1451) ng/mL in the control group, expressed in the median (25th percentile, 75th percentile) (Supplementary Table S1). The results showed that serum MBL concentration in the obese group was significantly lower than that of the control group, (*p* < 0.001, Figure 1A). In the obese group, we further measured the cIMT, lipid profile, blood pressure, hip circumference (HC), and waist circumference (WC), and then investigated the risk factor of cIMT. We found that blood glucose was positively corelated with both right and left cIMT, and weight and BMI were positively correlated with right cIMT, while not significantly in left cIMT. The serum MBL concentration was negatively correlated with both right and left cIMT, although the correlation was not strong compared with blood glucose, but the difference was significantly (*p* < 0.05, Figure 1B).

### 3.2. Autophagy Is Involved in ox-LDL-Induced EC Injury, Proliferation, and Apoptosis

**Figure 2:**
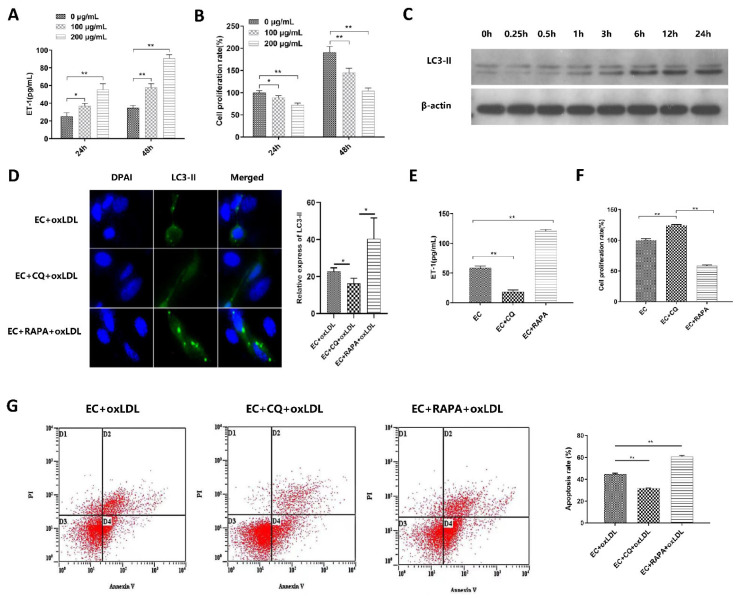
**Ox-LDL-induced EC autophagy and injury (*n* = 3).** (**A**) ET-1 level in ECs after treatment with different concentration of ox-LDL and time durations: the results showed that the ET-1 level which reflected ECs damage was increased with ox-LDL concentration and time duration. (**B**) EC proliferation rate after treatment with different concentration of ox-LDL and time durations: the results revealed that EC proliferation rate was decreased with ox-LDL concentration and time duration. (**C**) The expression level of LC3-II in 100 μg/mL ox-LDL treatment for different time durations: 100 μg/mL ox-LDL treatment for 24 h significantly increased the expression of LC3-II. (**D**) Fluorescence expression of LC3-II protein in ECs in each group: LC3-II-GFP expression was significantly reduced in the CQ-treated group, while increased in the RAPA-treated group (400 times). (**E**) ET-1 level in ECs of each group: ET-1 level was significantly reduced in the CQ treated group, while increased in the RAPA treated group. (**F**) EC proliferation rate of each group: the proliferation rate of ECs was significantly increased in the CQ treated group, while reduced in the RAPA treated group. (**G**) Flow diagram of EC apoptosis: EC apoptosis rate was significantly reduced in the CQ treated group, while increased in the RAPA treated group. Note: CQ is chloroquine, and RAPA is rapamycin. Ox-LDL means 100 μg/mL ox-LDL treatment for 24 h; CQ (25 μM) is pretreated for 1 h, and then treated with ox-LDL; RAPA (0.02 μg) is pretreated for 1 h, and then treated with ox-LDL. ** *p* < 0.01, * *p* < 0.05.

Vascular ECs were treated with ox-LDL (0, 100, 200 μg/mL) for 24 h and 48 h. ET-1 level, the marker of ECs damage, was significantly increased while the EC proliferation rate was decreased with ox-LDL concentration and treated time duration (Figure 2A,B). The results showed that ox-LDL-induced EC injury in both a time and dose-dependent manner. Treatment with 100 μg/mL ox-LDL for 24 h significantly increased the LC3-II protein expression in ECs compared with 0 h (Figure 2C). Thus, 100 μg/mL ox-LDL treatment for 24 h was utilized in the subsequent experiments.

ECs were pretreated with autophagy regulators chloroquine and rapamycin to investigate the role of autophagy in ox-LDL-induced EC injury. After treatment with autophagy inhibitor chloroquine (CQ) and autophagy inducer rapamycin (RAPA), the LC3-II-GFP expression (Figure 2D), ET-1 level (Figure 2E), and EC apoptosis rate (Figure 2G) were significantly reduced in the CQ treated group, while increased in the RAPA treated group. However, the proliferation rate of ECs (Figure 2F) was significantly increased in the CQ treated group, while reduced in the RAPA treated group compared with the control group. These data suggest that autophagy is involved in ox-LDL-induced EC injury, proliferation, and apoptosis.

### 3.3. Ox-LDL-Induced EC Autophagy and Injury via LOX1 Pathway

**Figure 3:**
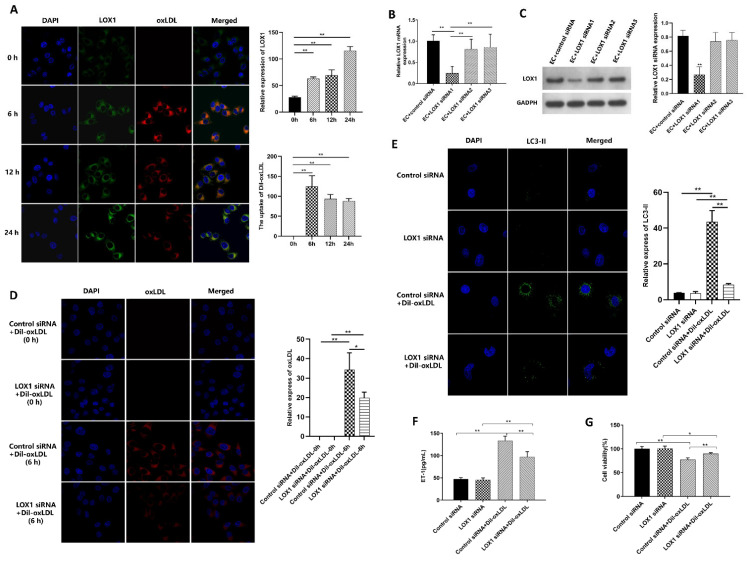
**The role of LOX1 in ox-LDL-induced EC autophagy (*n* = 3).** (**A**) The localization of LOX1 and ox-LDL in ECs treated with Dil-ox-LDL at different time points (200 times). (**B**) LOX1 mRNA expression levels of ECs in each group: LOX1-siRNA was transfected into ECs, and LOX1 mRNA was significantly downregulated compared with control group. (**C**) LOX1 protein expression level of ECs in each group: after the transfection of LOX1-siRNA, the expression of LOX1 protein was significantly decreased compared with the control group. (**D**) The localization of ox-LDL in the ECs at 0, 6 h of Dil-ox-LDL treatment (200 times): the red fluorescence reflecting the uptake of Dil-ox-LDL, was significantly decreased after LOX1 knock-down. (**E**) Immunofluorescence of LC3-II (200 times): LC3-II was measured in ECs with or without LOX1 knock-down groups, and the green fluorescence was significantly decreased in the LOX1-siRNA group. (**F**) ET-1 level in the supernatant of ECs in each group: ET-1 level was significantly decreased in the LOX1-siRNA group compared with the control siRNA group after ox-LDL treatment. (**G**) Cell viability of ECs in each group: cell viability was significantly increased in the LOX1-siRNA group compared with the control siRNA group after ox-LDL treatment. Note: ** *p* < 0.01, * *p* < 0.05. Control siRNA means using control siRNA to transfect ECs in the upper chamber and moving to a well plate containing ECs after 48 h; LOX1-siRNA means using LOX1-siRNA to transfect ECs in the upper chamber and moving to containing ECs after 48 h in a well plate; Dil-ox-LDL means 100 μg/mL Dil fluorescently labeled ox-LDL.

ECs were treated with Dil fluorescently labeled ox-LDL. The uptake of Dil-ox-LDL was coincided with LOX1 expression, and the uptake of Dil-ox-LDL was significantly increased at 6 h but did not increase with time, while the expression of LOX1 was strongest at 24 h; thus, 6 h was selected as the detection time (Figure 3A). To investigate the role of LOX1 in ox-LDL-induced EC autophagy, LOX1-siRNA and control siRNA were transfected into ECs, and LOX1 mRNA and protein were significantly down-regulated in the LOX1-siRNA group (Figure 3B,C).

After Dil-ox-LDL treatment, the red fluorescence (Figure 3D) and LC3-II aggregation (Figure 3E) were significantly reduced in the LOX1-siRNA group compared with the control siRNA group. The ET-1 level was significantly decreased (Figure 3F) and the activity of ECs increased (Figure 3G) in the LOX1-siRNA group compared with the control siRNA group. These findings suggest that ox-LDL-induced EC autophagy and injury via LOX1 and down-regulating LOX1 can alleviate ox-LDL-induced EC injury.

### 3.4. MBL Over-Expression In Vitro May Block the Binding of LOX1 and ox-LDL, Further Inhibiting ox-LDL-Induced EC Autophagy and Injury

**Figure 4:**
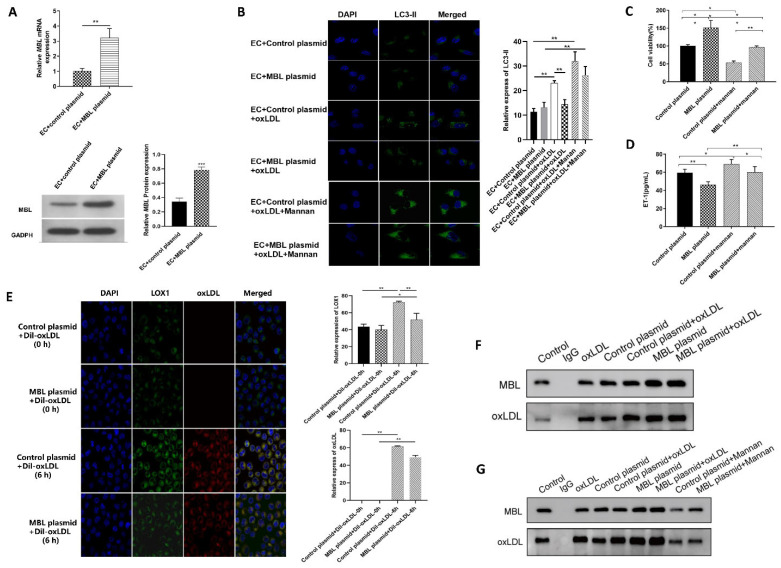
**MBL reduce ox-LDL-induced EC injury in vitro (*n* = 3).** (**A**) The expression of MBL mRNA and protein in transfected ECs: MBL over-expressed plasmid was constructed and transfected into ECs, and the expression of MBL mRNA and protein were significantly increased compared with the control group. (**B**) The expression of LC3-II in ECs of each group (200 times): The green fluorescence of LC3-II was significantly increased in the ox-LDL treatment group, while the MBL plasmid transfected group reduced the expression of LC3-II in ox-LDL-treated ECs compared with the control plasmid group. MBL plasmid transfected ECs were incubated with mannan, and then treated with ox-LDL; the green fluorescence of LC3-II significantly increased compared with the group without mannan treatment. (**C**) EC viability detection in each group: The viability of ox-LDL-treated ECs was increased after being transfected with MBL plasmid, while decreased after mannan treatment. (**D**) ET-1 level of EC supernatant measured by Elisa: The ET-1 level of ox-LDL-treated ECs was decreased after being transfected with MBL plasmid, while increased after mannan treatment. (**E**) The localization of LOX1 and ox-LDL in ECs treated with Dil-ox-LDL for different times (200 times): After Dil-ox-LDL treatment for 6 h, the red fluorescence of ox-LDL and the green fluorescence of LOX1 were increased in both groups compared with 0 h, while both red and green fluorescence were decreased in MBL plasmid group compared with control plasmid group. (**F**) Co-immunoprecipitation bands of transfected ECs: Both ox-LDL and MBL protein expressions were significantly increased in the MBL plasmid group compared with the control plasmid group. (**G**) Co-immunoprecipitation bands of transfected ECs treated with mannan: After mannan treatment, the expression of ox-LDL and MBL protein decreased in both MBL plasmid and control plasmid groups. Note: * *p* < 0.05, ** *p* < 0.01, *** *p* < 0.001. EC + control/MBL plasmid means ECs were transfected with control/MBL plasmid and incubated for 48 h; EC + control/MBL plasmid + ox-LDL indicate transfected ECs incubated for 48 h, and then treated with 100 μg/mL ox-LDL for 24 h. EC + control/MBL + ox-LDL + mannan means that transfected ECs incubated with mannan for 48 h, and then treated with 100 μg/mL ox-LDL for 24 h.

MBL over-expressed plasmid was constructed, and the expression of MBL mRNA and protein in ECs significantly increased after transfection with MBL over-expression plasmid (Figure 4A). After ox-LDL treatment, LC3-II aggregation was significantly reduced in the MBL plasmid transfected group compared with the control plasmid group (Figure 4B). Mannan was used to competitive binding with MBL, and the aggregation of LC3-II significantly increased after treatment with mannan compared with the group without mannan (Figure 4B). Moreover, mannan also reversed the protective effect of MBL on EC viability (Figure 4C) and injury (Figure 4D). These data confirm that MBL can protect ECs from ox-LDL-induced autophagy and cell damage, and that mannan can reverse the protective effect of MBL on ECs.

To further explore the mechanism of MBL in protecting ECs from ox-LDL-induced injury, we performed immunofluorescence localization of LOX1 and ox-LDL in ECs. After Dil–ox-LDL treatment for 6 h, the red fluorescence of ox-LDL and green fluorescence of LOX1 increased in both groups compared with 0 h, indicating that ECs can uptake ox-LDL, and ox-LDL promotes the expression of LOX1 (Figure 4E). Compared with the control plasmid group, the green fluorescence of LOX1, the red fluorescence of ox-LDL, and the overlap between LOX1 and ox-LDL were reduced in the MBL plasmid group, indicating that MBL over-expression may block the combination of LOX1 and ox-LDL. Further co-immunoprecipitation showed that both ox-LDL and MBL protein expressions were significantly increased in the MBL plasmid group compared with the control plasmid group (Figure 4F). However, compared with the MBL Plasmid group, ox-LDL protein expression was decreased after mannan treatment, indicating that mannan could reduce the interaction between ox-LDL and MBL (Figure 4G). These data suggested that MBL may block the binding of LOX1 and ox-LDL in vitro, thus further inhibiting ox-LDL-induced EC autophagy and injury.

### 3.5. MBL Alleviates Atherosclerotic Plaque Formation by Modulating Autophagy and Competitively Inhibiting LOX1-ox-LDL Binding

An MBL over-expression adenovirus was constructed (Supplementary Figure S1A–D). Different concentrations of the MBL over-expression adenovirus were administered with retro-orbital injections into ApoE^−/−^ mice. The expression of MBL in mice serum was significantly increased after 2 weeks of injection and began to decrease after 4 weeks. In addition, there was no significant difference between 1 × 10^9^ PFU/mouse and 2 × 10^9^ PFU/mouse of MBL adenovirus injection (Supplementary Figure S1E). Consequently, 1 × 10^9^ PFU/mouse of MBL/control adenovirus with injection every 4 weeks was selected for the study.

**Figure 5:**
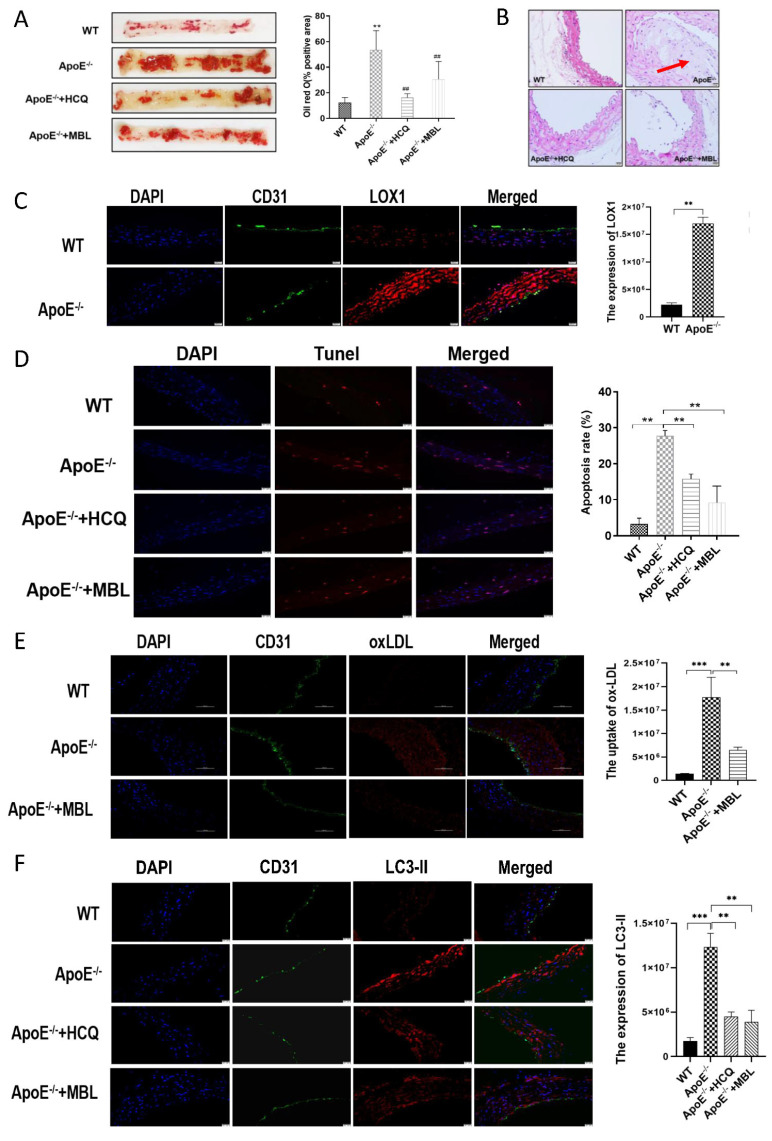
**Experimental findings in vivo (*n* = 5).** Vascular endothelial tissue of the thoracic aorta from mice fed with 16 weeks’ HFD was collected for the following experiments, and CD31 was used to label the vascular endothelium. (**A**) Oil red O staining: the lipid deposition was significantly increased in ApoE^−/−^ mice after 16 weeks of HFD compared with WT mice; lipid deposition was alleviated in mice injected with autophagy inhibitor chloroquine and MBL adenovirus. (**B**) HE staining: ApoE^−/−^ mice showed marked atherosclerotic plaques after 16 weeks of HFD (see the red arrow), while the atherosclerotic plaques disappeared in mice injected with chloroquine and MBL adenovirus; the scale bar indicates 20 μm. (**C**) LOX1 expression in ECs: The red fluorescence of LOX1 in ECs was significantly increased in ApoE^−/−^ mice after 16 weeks of HFD compared with WT mice; the scale bar indicates 20 μm. (**D**) Apoptosis rate of ECs in each group: TUNEL staining was used to measure the apoptosis of ECs from thoracic aorta. The red fluorescence was significantly increased in ApoE^−/−^ compared with WT mice after 16 weeks of HFD, while decreased significantly after chloroquine and MBL-adenovirus injection; the scale bar indicates 20 μm. (**E**) Uptake of ox-LDL in each group: the red fluorescence of ox-LDL was significantly increased in ApoE^−/−^ compared with WT mice after 16 weeks of HFD, and decreased significantly after MBL-adenovirus injection; the scale bar indicates 50 μm. (**F**) LC3-II expression in each group: the red fluorescence of LC3-II was significantly increased in ApoE^−/−^ compared with WT mice after 16 weeks of HFD, while decreased significantly after chloroquine and MBL-adenovirus injection; the scale bar indicates 50 μm. Note: ** *p* < 0.01, *** *p* < 0.01, ## *p* < 0.01.

ApoE^−/−^ mice fed with HFD were used to establish atherosclerosis model. Intraperitoneal chloroquine injections were performed combined with HFD in ApoE^−/−^ mice every day. After 16 weeks, vascular endothelial tissues of the thoracic aorta were collected and stained with Oil Red O, HE, TUNEL staining, and immunohistochemical. The results showed that the lipid deposition (Figure 5A), atherosclerotic plaques (Figure 5B), LOX1 expression (Figure 5C), apoptosis rate (Figure 5D), uptake of ox-LDL (Figure 5E), and the expression of LC3-II (Figure 5F) in ECs of aortas were significantly increased in ApoE^−/−^ mice compared with WT mice. Chloroquine injection can revise ox-LDL-induced EC autophagy and injury. Interestingly, MBL over-expression in vivo could significantly reduce fat deposition, the atherosclerotic plaques, the apoptosis rate, the uptake of ox-LDL, and the expression of LC3-II in mice aorta ECs compared with the control group. These data showed that HFD could increase the expression of LOX1 and uptake of ox-LDL and aggravate the formation of atherosclerotic plaques, and MBL can alleviate atherosclerotic plaque formation by modulating autophagy and competitively inhibiting LOX1-ox-LDL binding.

### 3.6. MBL Alleviates ox-LDL-Induced ECs Injury and Atherosclerotic Plaque Formation via Modulating DCs Matsuration

**Figure 6:**
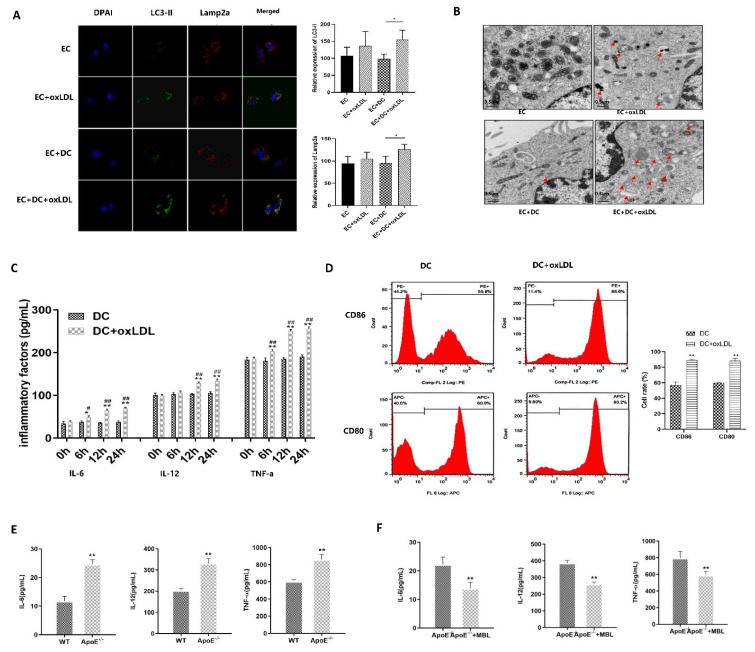
**Role of MBL and DCs in ox-LDL-induced EC injury and atherosclerotic plaque formation (*n* = 3).** (**A**) Co-localization of LC3-II and Lamp2a in each group (400 times): DCs were co-cultured with ECs, ox-LDL treatment increased the co-localization of LC3-II and Lamp2a, and the green fluorescence of LC3-II and the red fluorescence of Lamp2a were significantly increased in the DC co-culture group. (**B**) Electron microscopy of autophagosomes in each group: Autophagosomes were indicated by red arrows, ox-LDL treatment increased the formation of autophagosomes in ECs, and DC co-culture further increased the formation of autophagosomes in ECs after ox-LDL treatment. (**C**) The expression level of inflammatory factors in the supernatant of DCs treated with ox-LDL: The expression of IL-6, IL-12, and TNF-a was up-regulated with time in DCs after treatment with ox-LDL. (**D**) DC maturation detection: Flow cytometric analysis was used to measure the maturation of DCs; the expression of CD86 and CD80 was significantly increased after ox-LDL treatment. (**E**) Serum levels of inflammatory factors in ApoE^−/−^ and WT mice: The serum levels of IL-6, IL-12, and TNF-a were significantly increased in ApoE^−/−^ mice compared with WT mice. (**F**) Serum levels of inflammatory factors in MBL transfected ApoE^−/−^ and ApoE^−/−^ mice: Serum levels of IL-6, IL-12, and TNF-a were significantly decreased in MBL-adenovirus compared to blank-adenovirus injected ApoE^−/−^ mice. * *p* < 0.05, ** *p* < 0.01. In (**C**), * means the difference expression of inflammatory factors between DC and DC + ox-LDL group in each time point, # means the difference expression of inflammatory factors in each time point compared to 0 h in DC + ox-LDL group, ## *p* < 0.01.

In vitro, DCs were co-cultured with ECs, and the co-localization of LC3-II and Lamp2a in ECs increased in the DC co-culture group (Figure 6A). Electron microscopy also showed that DC co-culture further promoted autophagosome formation in ECs (Figure 6B). When DCs were treated with ox-LDL, the expression of IL-6, IL-12, and TNF-a was up-regulated in a time-dependent manner (Figure 6C). Flow cytometric analysis revealed that the expression of CD86 and CD80 was significantly increased after the treatment of ox-LDL compared with the group without ox-LDL (Figure 6D). These data suggest that ox-LDL treatment can induce DC inflammation and maturation, and DC co-culture further promotes ox-LDL-induced EC autophagy.

In vivo, ApoE^−/−^ mice fed with HFD for 16 weeks, and MBL over-expression adenovirus was injected retro-orbital every 4 weeks, then the spleen was collected for DC isolation, and serum was collected for inflammatory factors detection. Flow cytometry analysis showed that the proportion of CD11c + CD80+ and CD11c + CD86+ in spleen-derived DCs of ApoE^−/−^ mice were significantly higher than that of WT mice (Supplementary Figure S2). In addition, the serum levels of IL-6, IL-12, and TNF-a were significantly increased (Figure 6E). However, DC maturation rate (Supplementary Figure S3) and serum levels of IL-6, IL-12, and TNF-a (Figure 6F) were significantly decreased in the MBL over-expression group compared to blank-adenovirus injected ApoE^−/−^ mice. These data indicated that MBL overexpression in vivo could influence the maturation of DCs and down-regulate inflammation, which may eventually alleviate EC injury and the progression of atherosclerosis.

## 4. Discussion

CVDs has become the leading cause of death and disability worldwide, and atherosclerosis is a major contributor. Low-density lipoprotein (LDL) accumulation is the initiating factor of atherosclerosis, and the subsequent inflammatory response contributes to disease progression and outcome. MBL, the key molecule in the innate immune system, has played a role in complementing activation [23], promoting the complement-independent opsonophagocytosis [24] and clearance of apoptotic cells [25]. Recently, MBL was also found to participant in adaptive immune responses, and involved in insulin resistance, diabetes, atherosclerosis, and myocardial infarction [26,27]. The current study reported a protective role of MBL in ox-LDL-induced EC injury in vivo and in vitro. The main findings of this study are as follows: (1) Serum MBL concentration was decreased in children with obesity and negatively correlated with cIMT. (2) Autophagy is involved in ox-LDL-induced EC injury via the LOX1 pathway. (3) MBL can modulate EC autophagy and DC maturation, further inhibiting ox-LDL-induced atherosclerosis. These data confirm the role and potential mechanisms of MBL in protecting against CVDs and provide direction for drug intervention.

The three missions of autophagy include defense, metabolic, and quality control, which are the bridge linking innate immunity and adaptive immunity [28]. Previous studies have revealed that proper autophagy is beneficial in atherosclerosis [29,30], while excessive autophagy is detrimental to CVDs [31,32]. As an important complement molecule in the innate immune system, MBL is also a key soluble pattern recognition receptor. Whether MBL can regulate autophagy was unclear. Study has reported that MBL can accelerate the autophagy process and reduce lipid accumulation during 3T3-L1 adipocyte differentiation through AMPK/mTOR signaling pathway [33]. Therefore, we hypothesized that MBL could regulate autophagy and participate in the formation and progression of atherosclerotic plaque. Firstly, we measured serum MBL concentration in children with obesity and found that MBL level decreased in obese children compared with healthy controls, and lower MBL concentration indicated increased cIMT. To further explore the underlying mechanism of MBL in ECs injury, ECs were treated with ox-LDL, and the expression of LC3-II was found to increase in a dose- and time-dependent manner (Figure 2C). Autophagy modulators were applied to further confirm the involvement of autophagy in ox-LDL-induced EC injury, the expression of LC3-II, and EC damage marker ET-1, and the EC apoptosis rate was decreased in the autophagy inhibitor chloroquine pretreated group, while increased in the autophagy inducer rapamycin pretreated group (Figure 2D,E,G). We then constructed MBL over-expression plasmid, and mannan was used for competitive inhibition of MBL. The results showed that MBL over-expression decreased the expression of LC3-II, while mannan can specifically bind to MBL and finally increase the expression of LC3-II (Figure 4B), which indicated that MBL could reduce ox-LDL-induced ECs autophagy in vitro study. For in vivo study, WT and ApoE^−/−^ mice were fed with HFD for 16 weeks, endothelial tissue from the thoracic aorta was collected; the lipid deposition and atherosclerotic plaque formation in HFD ApoE^−/−^ mice significantly increased in ApoE^−/−^ mice compared with WT mice, while chloroquine injection reduced endothelial lipid deposition, plaque formation, and cell apoptosis (Figure 5A,B,D). These data reveal that autophagy was involved in HFD-induced atherosclerosis. We constructed MBL over-expression adenovirus, and over expression of MBL in vivo also reduced endothelial lipid deposition, plaque formation, and cell apoptosis, and meanwhile decreased the expression of LC3-II (Figure 5A,B,D,F). Thus, we concluded that MBL protects ECs from ox-LDL-induced injury by modulating autophagy both in vivo and vitro.

Ox-LDL was mainly up-taken by ECs via binding with LOX1. Several studies have shown the vital role of LOX1 in ox-LDL-induced EC autophagy and injury [12,13]. Coincidentally, both LOX1 and MBL belong to the C-type lectin family, and MBL could bind to ox-LDL in vitro. Therefore, we speculated that MBL might inhibit the binding of ox-LDL to LOX1. In vitro, LOX1 was knocked down by siRNA, and down-regulation of LOX1 could inhibit the uptake of ox-LDL, alleviate EC autophagy and injury, and increase EC viability compared with the control siRNA group (Figure 3D–G). Co-IP revealed that ox-LDL protein expression was significantly increased after ox-LDL and MBL overexpressed plasmid treatment compared with the control group, and mannan can decrease the expression of ox-LDL protein, which confirmed the protein interaction between ox-LDL and MBL in ECs (Figure 4F,G). In the in vivo study, LOX1 expression increased in HFD ApoE^−/−^ mice, while MBL over-expression reduced LOX1 expression and ox-LDL uptake (Figure 5C,E). These data suggest that MBL ameliorates EC injury via modulating autophagy by blocking the binding of ox-LDL and LOX1.

Numerous studies have focused on the contributions of innate and adaptive immune cells, particularly DCs, macrophages, and T cells, in atherosclerosis progression [5]. MBL could attenuate peptidoglycan-induced inflammatory cytokine production and suppress nuclear translocation of NF-κBp65 [34]. High concentrations of MBL can inhibit the differentiation of DCs and attenuate LPS-induced maturation of DCs [35,36]. In this study, we found that ox-LDL-treated in vitro or HFD-fed mice in vivo could induce DC maturation and increase the production of inflammation factors IL-6, IL-12, and TNF-a. DC co-culturing with ECs can promote ox-LDL-induced EC autophagy. In addition, MBL over-expression in ApoE^−/−^ mice could reduce the secretion of inflammation factors, as well as affect DC maturation. These data provide further support for the vital role of inflammation in the development of atherosclerosis and provide evidence that MBL may modulate DC maturation and inflammation response. Although the protective or detrimental role of MBL in CVDs is still controversial, as both low and high levels of MBL indicate the susceptibility to CVDs [19,20], our findings provide solid evidence that MBL can be potentially used for immune-modulatory therapy for atherosclerosis.

There are still limitations in our study. First, we utilized LOX1-siRNA to investigate the role of LOX1 in ECs injury in vitro, while we did not construct LOX1 knockout mice for financial reasons. Second, the autophagy analysis was not optimal; bafilomycin A1 should be used to distinguish the increase in autophagosome formation from a decrease of autophagosome degradation. Thirdly, we did not quantitatively investigate the role of MBL in regulating EC injury. Fourth, we know that lifestyles including diet and physical activity are crucial for preventing CVDs, so whether these confounders interacted with MBL should be investigated. Physical activity may have a more important role in CVDs than we expected before; on the one hand, physical activity can directly regulate immune cells, cytokines, and inflammatory responses, and on the other hand, it may affect the composition of the microbiome, and the microbiome may act as a regulator of inflammation in the progression of metabolic diseases [37,38]. A recent study reported the gut microbiota structurally changed in MBL-deficient mice, and the function prediction revealed that the abundance of predicted genes in immune system and metabolic disease pathways were significantly increased in MBL^−/−^ mice [39]. Thus, more longitudinal cohort clinical trials and animal studies are needed to investigate the role of MBL and the interaction with the microbiome and the immune regulator in CVDs, as well as to determine the optimal level of MBL for CVD patients.

## 5. Conclusions

This study showed a protective role of MBL in ox-LDL-induced EC injury. To the best of our knowledge, this is the first study that has reported that MBL could protect ECs through modulating autophagy by competitive inhibition of the ox-LDL and LOX1 binding. We also found that MBL may modulate DC maturation and inflammation response, providing potential use for MBL in immune-modulatory therapy for atherosclerosis.

## Data Availability

We did not report any data.

## Supplementary Materials

The following supporting information can be downloaded at: https://www.mdpi.com/article/10.3390/biomedicines11061743/s1, Figure S1: Identification of adenovirus packaging; Figure S2: DCs maturation rate in ApoE^−/−^ and WT mice; Figure S3: DCs maturation rate in MBL transfected ApoE^−/−^ and ApoE^−/−^ mice; Table S1: Clinical characteristics and serum MBL concentration of children with obesity and healthy controls.

## Abbreviations

MBL: mannose-binding lectin; ECs: endothelial cells; ox-LDL: oxidized low-density lipoprotein; LOX1: lectin-like oxidized low-density lipoprotein receptor-1; DCs: Dendritic cells; CCK-8: Cell Counting Kit-8; CVDs: cardiovascular diseases; CQ: Chloroquine; RAPA: rapamycin; siRNA: small interfering RNA; Co-IP: Co-Immunoprecipitation.
