# Supplementary material for: Mannose-Binding Lectin Reduces Oxidized Low-Density Lipoprotein Induced Vascular Endothelial Cells Injury by Inhibiting LOX1-ox-LDL Binding and Modulating Autophagy
Source: Biomedicines. 2023 Jun 17;11(6):1743. doi: 10.3390/biomedicines11061743 (PMC10296346; doi:10.3390/biomedicines11061743)
Supplement: Supplementary file 1 [file biomedicines-11-01743-s001.zip › biomedicines-2315465-supplementary.pdf]

Supplementary Table

Table S1. Clinical characteristics and serum MBL concentration of children with obesity and healthy controls (n =199)

Characteristics	Overall	Control group	Obese group	P
Number	199	105	94	
Male (%)	65.69	58.10	73.74	
Age (y), mean (SD)	10.36(2.62)	10.35(2.51)	10.38(2.74)	0.56
BMI (kg/m2), median (IQR)	23.46(8.75)	19.06(2.82)	28.12(5.12)	<0.001
ALT (U/L), median (IQR)	38.47(20.50)	21.54(13.00)	56.42(53.00)	<0.001
AST (U/L), median (IQR)	33.79(14.00)	29.53(12.50)	38.31(23.00)	<0.001
MBL (ng/ml), median (IQR)	927.60(908.59)	909.69(523.51)	625.90(1151.85)	<0.001
SBP (mmHg), mean (SD)			149.99(14.38)	
DBP (mmHg), median (IQR)			66.00(11.00)	
TG (mmol/L), median (IQR)			1.12(0.64)	
TC (mmol/L), mean (SD)			4.21(0.47)	
LDL-C(mmol/L), mean (SD)			2.44(0.55)	
HDL-C(mmol/L), mean (SD)			1.24(0.25)	
Left cIMT(cm), median (IQR)			0.06(0.02)	

Right cIMT(cm), median (IQR)	0.06(0.01)
HC (cm)	93.05(10.04)
WC (cm)	95.60(10.42)

Supplementary figures

A

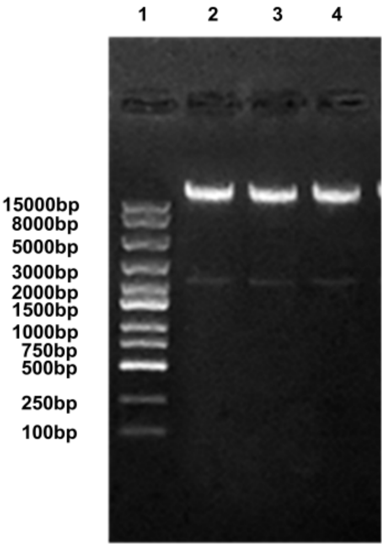

1.15000maker:15000bp,8000bp,5000bp,3000bp,20
1000bp,750bp,500bp,250bp,100bp
2.pAd-pYr-ads-1-MCS-MBL-1（Pac I）
3.pAd-pYr-ads-1-MCS-MBL-2（Pac I）
4.pAd-pYr-ads-1-MCS-MBL-3（Pac I）

B

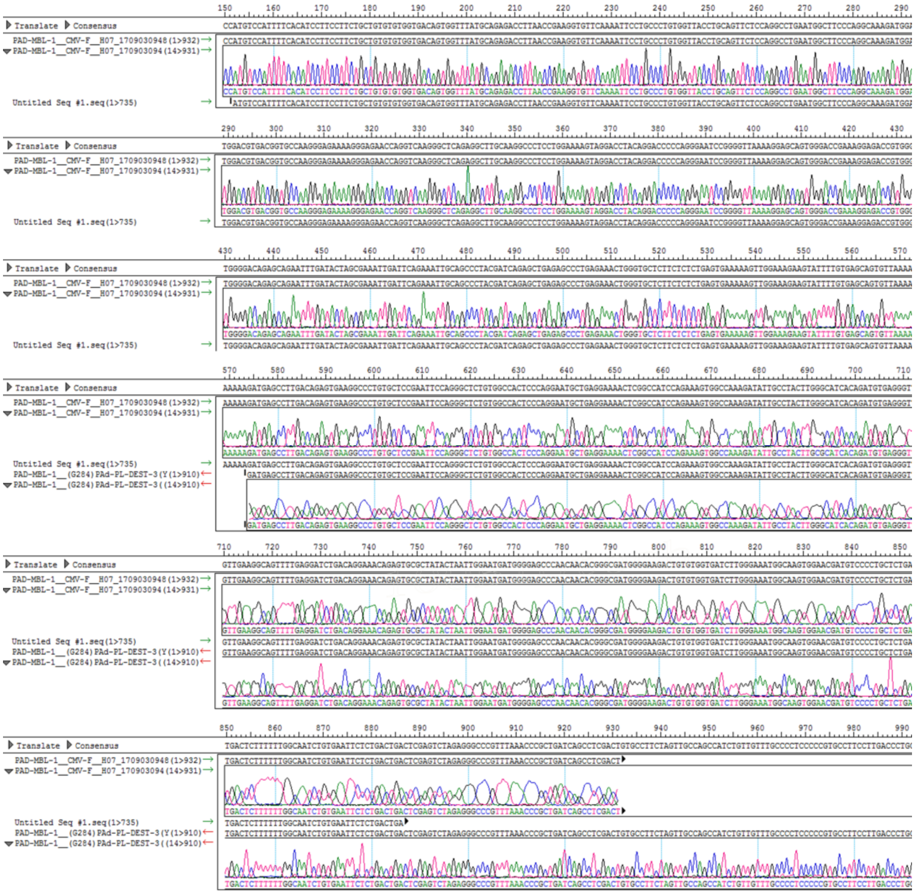

C

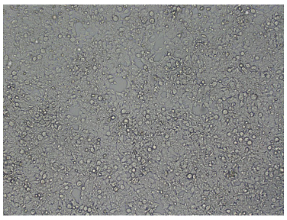

D

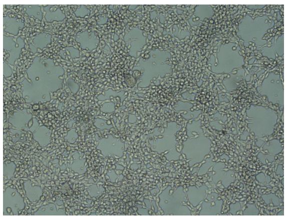

E

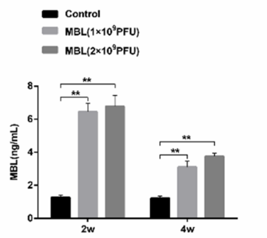

Figure S1 **Identification of adenovirus packaging.** A. Enzyme digestion identification map. B. Sequencing identification. C. White light image at 48 h after adenovirus infection. D. The white light image after amplification. E. MBL level in serum. **Indicates $p<0.01$ vs. WT or control, ##Indicates $p<0.01$ vs. $ApoE^{-/-}$.

A

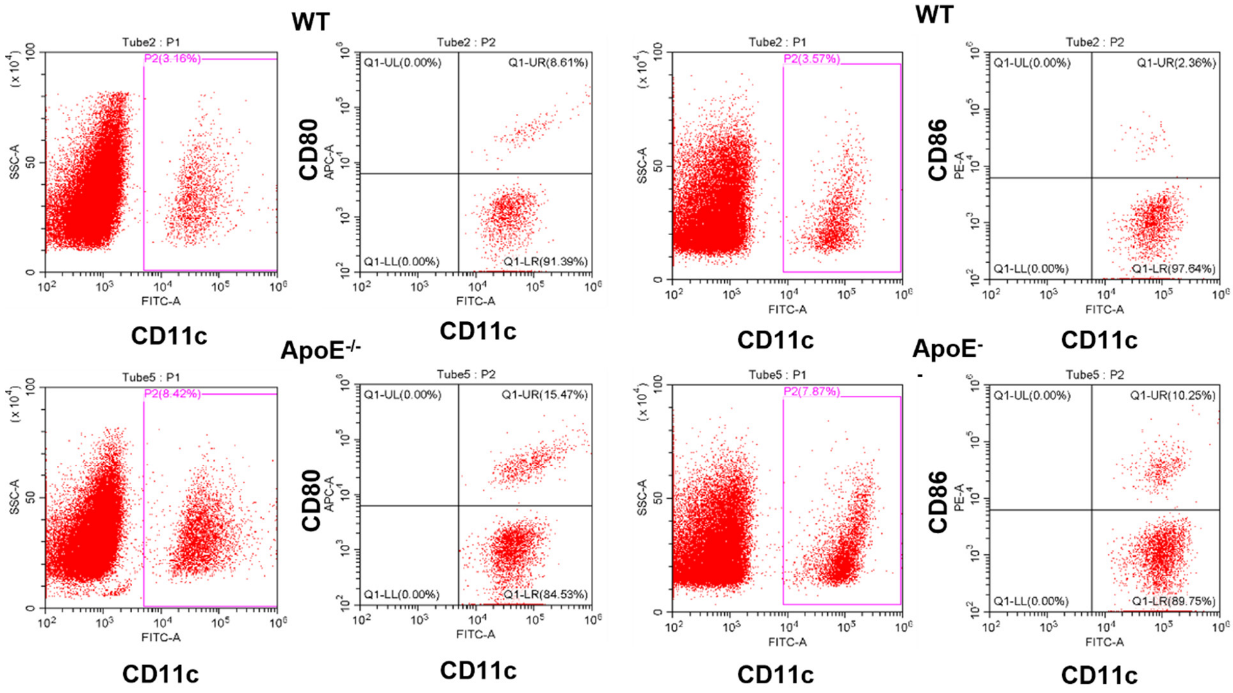

B

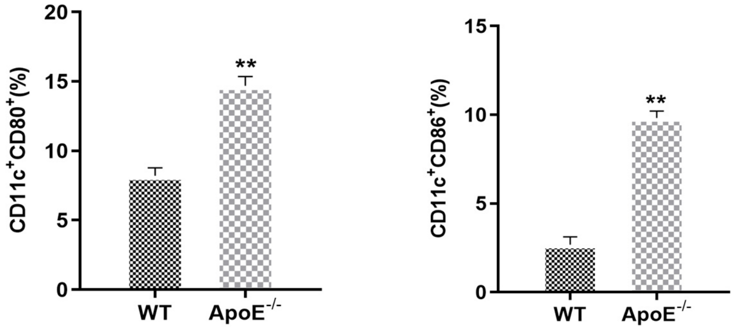

Figure S2. DCs maturation rate in ApoE$^{-/-}$ and WT mice. DCs were isolated from the spleen of ApoE$^{-/-}$ and WT mice after 16 weeks' HFD. A. Fow cytometry of DCs, B. Quantification of DCs: the proportion of CD11c+CD80+ and CD11c+CD86+ in ApoE$^{-/-}$ mice were significantly higher than that of WT mice. Note: ** $p < 0.01$.

A

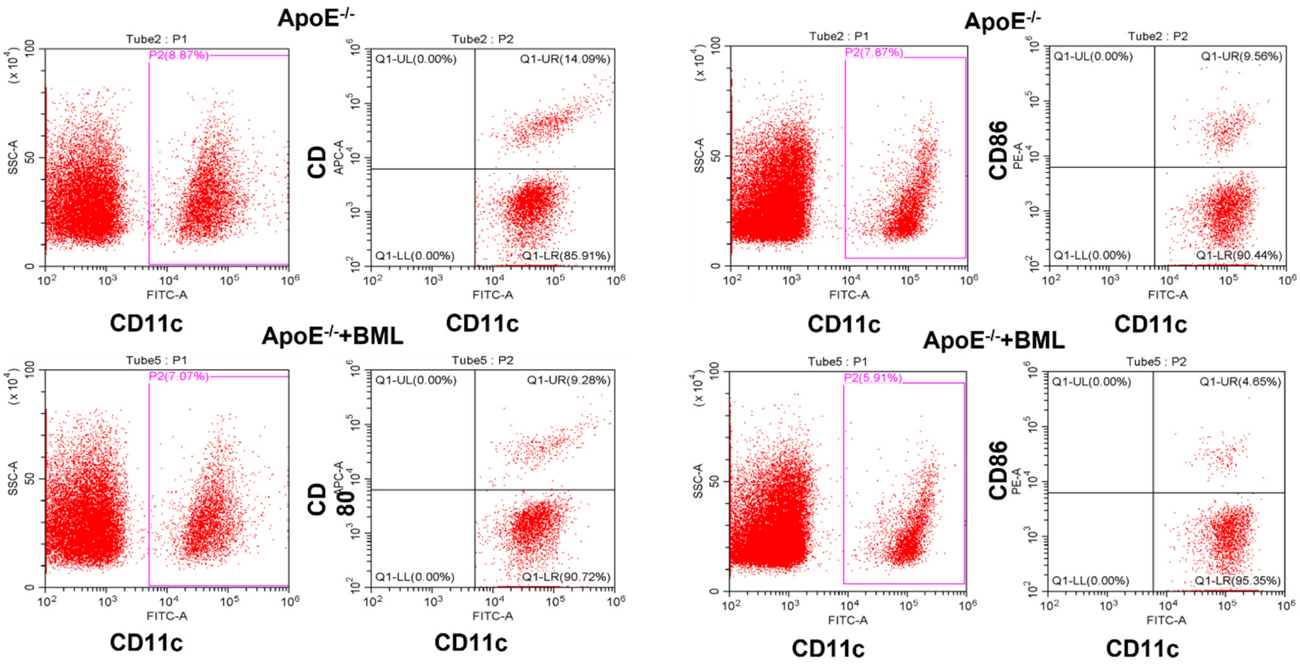

B

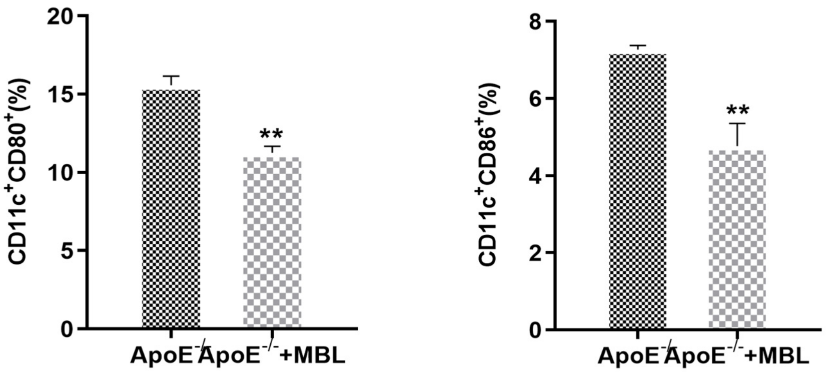

Figure S3. DCs maturation rate in MBL transfected ApoE$^{-/-}$ and ApoE$^{-/-}$ mice. DCs were isolated from the spleen of ApoE$^{-/-}$ and WT mice after 16 weeks' HFD. A. Flow cytometry of DCs, B. Quantification of DCs: the proportion of CD11c+CD80+ and CD11c+CD86+ were significantly decreased in MBL-adenovirus compared to blank-adenovirus injected ApoE$^{-/-}$ mice. Note: ** $p < 0.01$.
